# Long-term storage under pressure in deep sea improved the microbiological safety and physical properties of whale meat

**DOI:** 10.1016/j.heliyon.2024.e29631

**Published:** 2024-04-12

**Authors:** Satomi Tsutsuura, Maki Matsumoto, Kana Sakai, Ryosuke Motegi, Tadayuki Nishiumi

**Affiliations:** Faculty of Agriculture, Niigata University, 8050 Ikarashi-2, Nishi-ku, Niigata, 950-2181, Japan

**Keywords:** Whale meat, Deep-sea storage, High pressure, Microbiological safety, Improvement in physical properties

## Abstract

This study aimed to clarify the effects of deep-sea pressure storage on the quality of whale meat, especially microbiological safety and physical properties, to examine the effectiveness of deep-sea storage for long-term aging of whale meat. Microbiological safety, physical properties, color and appearance, water content, water activity, and pH of whale meat were examined after storage in the deep sea at depths of 2200–6000 m (22–60 MPa) for 4 months. During storage under high pressure at a depth of >4000 m (40 MPa), the growth of aerobic bacteria was inhibited in whale meat. The toughness of whale meat stored in deep sea at a depth of >4000 m became significantly tender than that before deep-sea storage. Long-term storage of whale meat under high pressure and low-temperature conditions in the deep sea at a depth of >4000 m was clarified to improve the microbiological safety and tenderness of whale meat.

## Highlights

•During long-term storage under pressure, the growth of aerobic bacteria was inhibited in whale meat.
• The toughness of whale meat became significantly tender after long-term storage under pressure.
• Long-term storage at a depth of >4000 m in deep sea may be a suitable method for enhancing the quality and shelf life of food.

## 1 Introduction

Eating whales for a long time is a custom in Japan [[1], [2], [3]].^1^ During various periods, for example when eating meat was banned for religious reasons (around the year 675) and postwar food shortages (around 1945), whale meat has acted as a valuable source of animal protein for the Japanese population [4,5]. Whales are also traditionally mentioned in prayers for large catches and in celebrations; several aspects of whaling culture such as songs, dances, and traditional crafts persist to the present day [6,7]. In Japan, whaling is conducted in a manner that does not adversely affect whale stocks: the area of take is limited to only Japan's territorial waters and exclusive economic zone; only those whale species for which sufficient stocks have been confirmed, such as minke and sei whales are taken; and a quota below the International Whaling Commission's calculated take limit is set.^1^ As whales are large and consume huge quantities of fish, their numbers may affect the food security of coastal nations [8]. However, because whales are mammals and can only give birth to one or two calves for 2 years, the appropriate control of whaling is required [9,10]. As with other marine resources, the take and use of whales must be properly managed based on scientific evidence.

Whale meat is high in protein, but low in fat (Table 1) and cholesterol, and rich in iron [11,12].^2^ The main fats found in whale meat are polyunsaturated fatty acids, such as docosahexaenoic acid and eicosapentaenoic acid, which are rarely present in beef or other meats [13]. Whale meat is also characterized by its rich content of the free amino acid balenine, which is recognized for its antifatigue effects [14]. Although Japanese people today do not eat whale meat because of food shortage, as they did in the past, diets containing whale meat are expected to contribute to dietary diversity and maintain and improve health [15,16]. The consumption of whale meat is also expected to improve Japan's low food self-sufficiency ratio (currently approximately 39 %) and act as a substitute for meat, which is expected to become scarce in the future owing to the predicted global food crisis [17,18]. Although maintaining the culture and traditions of eating whale meat is a need, finding ways to process whale meat in a more palatable way as technology advances is necessary.

**Table 1:** Comparison of nutritional composition of whale meat, meat, and fish.^2^

Nutrient		Whale	Beef	Chicken	Tuna
Energy	(kcal)	100	176	234	115
Moisture	(g)	74.3	67.0	62.9	70.4
Protein	(g)	24.1	21.3	17.3	26.4
Fat	(g)	0.4	10.7	19.1	1.4
Carbohydrates	(g)	0.2	0.6	0.1	0.1
Ash	(g)	1.0	1.0	0.7	1.7
Salt Equivalent	(g)	0.2	0.1	0.1	0.1

Although several studies examining whale meat consumption and nutritional composition exist, the scientific knowledge of whale meat remains limited [[19], [20], [21]]. In general, whale meat is known to be elastic and fibrous, and to become even firmer after cooking, but the physical properties have not been measured. Compared with other meats, such as beef and chicken, whale meat is expected to be significantly firmer and is not necessarily appealing to consumers. Finding a way to improve the physical properties of whale meat is necessary for the practical application of whale meat processing and increasing its consumption.

High hydrostatic pressure (HHP) processing is one of the nonthermal processing technologies currently being exploited to produce value-added food products and maintain desirable sensory attributes [22]. When using HHP to process raw meat, there is a limit to the pressure that can be used, as undesirable effects on meat quality begin to occur at pressures above 150–200 MPa. Moreover, this level of pressure is not expected to effectively sterilize contaminating microorganisms in meat. Therefore, recently, the application of pressures below 100 MPa to food for extended periods has also been studied [[23], [24], [25], [26], [27], [28]] and is expected to have applications in food preservation, such as hyperbaric storage [23]. A few studies have reported the effects of pressure <100 MPa on microorganisms, color, and physical properties of raw meat [23]. In general, cell division is affected by pressure ranging between 20 and 50 MPa, and the growth of the microorganisms is restrained in a pressure range of 40–70 MPa depending on the species of microorganism [24,25]. The growth of spoilage bacteria was inhibited during cold storage of raw meat under pressure at 60 MPa for 60 days [26]. On the contrary, pressures <100 MPa were found to have little effect on meat color. The color of raw meat stored at 60 MPa for 60 days at a low temperature was the same as that stored at atmospheric pressure [26]. In previous studies of the physical properties [27,28], HHP treatment at 30–60 MPa was applied and the toughness of meat such as beef and pork softened only slightly. In these studies, raw meats were stored under high pressure for up to 14 days. However, the effect of pressure on the physical properties of raw meats during longer storage has not been investigated. Since the temperature and duration of HHP treatment significantly affect meat tenderization and pressure level, long-term storage under high pressure may reduce the toughness of whale meat. In addition, HHP processing equipment is expensive and increasingly cost inefficient: the longer the processing time, the fewer the samples can be processed [29]. Therefore, the long-term high-pressure processing of food is challenging with the current technologies available.

Thus, despite some studies on long-term storage under high pressure for foods, including raw meat, whale meat was not used. In addition, the effect of pressure on the toughness of whale meat, which is the most important factor to be improved in whale meat processing, has not been tested. The effects of high pressure on food quality may vary depending on the food type and should be examined using whale meat. For the application and practical use of long-term storage under high pressure in food processing, the effects of long-term pressure on contaminating microorganisms and important factors (physical properties, color and appearance, water content, etc.) that relate to quality must be examined.

In addition, food storage involves maintenance costs, such as refrigeration system and space. These factors will become important in establishing a sustainable method for longer storage. There are a few reports of attempts to store food in the deep sea, although meat has not been tested [30]. The effective utilization of unused resources could be achieved by using deep sea, with its low temperature variation, as a natural refrigerator, which will eventually contribute to the protection of the global environment.

This study aimed to clarify the effects of deep-sea pressure storage on the quality of whale meat, especially microbiological and physical properties, to examine the effectiveness of deep-sea storage for long-term aging of whale meat. Microbiological safety, physical properties, color and appearance, water content, water activity, and pH of whale meat before and after storage under high pressure and low-temperature conditions in the deep sea were examined. Changes in the quality of whale meat stored in the deep sea were also examined during refrigerated storage at atmospheric pressure, assuming the food processing and distribution.

## 2 Materials and methods

### 2.1 Sample preparation of whale meat and storage under pressure in deep sea

Red meat samples of sei whale (*Balaenoptera borealis*) from the Northwest Pacific Ocean were provided by Microbusto Japan Inc. (Tokyo, Japan). These meat samples were washed with cold water, cut into blocks of approximately 1 kg, vacuum-packed into polyethylene bags (Hiryu N-8, 180 × 260 cm, Asahi Kasei, Tokyo), and kept frozen until deep-sea storage. In total, 30 samples (equivalent to approximately 30 kg) were prepared. Five of these samples were analyzed as deep-sea prestorage samples. Deep-sea storage was performed using the following procedure based on the method of Hashimoto et al. [31]. Vacuum-packed samples were set on submergible equipment with mesh cages (TMSC-LM1111-GN, Trusco Nakayama Corporation, Tokyo, Japan) and mesh stages (TMSC-STD11-BK, Trusco Nakayama Corporation) [30]. The equipment was submerged by its weight with added iron plates. Fifteen samples were stored on the seafloor at depths of approximately 2200 m near the coast of Shizuoka, Japan, for 2 weeks, 3 months, and 4 months, in 2019 and 2020. After microbiological analysis was performed on samples stored at 2200 m, five samples were respectively stored on the seafloor at the depths of approximately 4000 and 6000 m near the coast of Ibaraki, Japan, for 4 months in 2021. Seawater temperature during deep-sea storage was recorded using a waterproof temperature logger (RBRsolo3 T Deep, RBR, Ottawa, Canada) located in the cage with samples. After the storage in the deep sea, the samples were transported frozen as well as the commercial food distribution and stored at −20 °C until use in the experiment.

### 2.2 Refrigerated storage test

In this test, whale meat samples before (control) and after deep-sea storage at depths of 2200 m (22 MPa), 4000 m (40 MPa) and 6000 m (60 MPa) and atmospheric pressure storage for 4 months, respectively were used. 1 kg of whale meat samples semi-thawed from −2 °C to 0 °C for 3 h were cut into blocks of approximately 60 g and vacuum-packed into polyethylene bags (Hiryu N-8, 180 × 260 cm, Asahi Kasei). The samples were stored at 4 °C for 60 days at atmospheric pressure for further analysis.

### 2.3 Microbiological tests

In the microbiological tests, total aerobic bacteria, total coliform, and *Vibrio parahaemolyticus* tests were conducted according to standard methods of analysis for food safety regulation [32] in Japan. Here, 25 g of raw whale meat was homogenized in 225 mL of sterilized buffered peptone water (BPW). Serial dilutions of the homogenate were prepared and cultured in Nutrient agar (Nissui Pharmaceutical, Tokyo) to enumerate total aerobic bacteria by the viable plate count method. The detection limits of the total aerobic bacteria were approximately 1.0 × 10^1^ CFU/g of whale meat. For the coliform test, the dilutions of the homogenate were cultured in desoxycholate agar (Eiken Chemical, Tokyo) and EMB agar (Eiken Chemical) to detect coliforms. Gas production of the isolates was confirmed in Lactose broth (Eiken Chemical) with Durhams tube, and colonial morphology was observed after gram staining. For *V. parahaemolyticus* test, whale meat samples were homogenized in nine volumes of 3 % NaCl-BPW. After dilution, it was inoculated in enrichment broth (alkaline peptone water) and incubated at 37 °C. The enriched broth was streaked on thiosulfate citrate bile salt sucrose (Eiken Chemical) agar plates, incubated, and observed for colonies. The density of *V. parahaemolyticus* was estimated using the most probable number method.

### 2.4 Texture profile analysis

To evaluate the effects of deep-sea storage on the texture of whale meat, the shear and compressive stress of whale meat were measured with a rheometer (Creep meter RE2-33005B, Yamaden, Tokyo) in accordance with the method described by Kim et al. [33] with modifications. For the measurement of shear and compressive stress, samples (approximately 40 g) were heated in a hot water bath at 80 °C for 30 min, cut into pieces measuring 10 × 10 × 10 mm, and then punctured with two types of plungers (wedge shape, 8 × 0.25 mm, 20 mm long; disk shape, 40 mm) at 5.0 mm/s, stopping at 100 % of the thickness. These experiments were conducted at room temperature (25 °C), and each sample was measured at least 10 times. The initial elastic modulus and compressive energy were calculated from the data obtained in the shear and compressive tests, respectively. The initial elastic modulus was calculated within the strain range of 0%–10 %.

### 2.5 Measurement of the color of whale meat

The color (L*, lightness; a*, redness; b*, yellowness) of the surface of raw whale meat was measured at four points using a color difference meter (CR-400, Konica Minolta, Tokyo) at room temperature (25 °C) in accordance with a previously reported method to measure meat color [33]. The ΔL*-, Δa*-, and Δb*-values of each sample between before and after refrigerated storage were calculated. The Chroma (C*)-values (saturation) and *ΔE*-values (color difference) were calculated using the following equation:

C* = [ (a*)^2^+(b*)^2^]^1/2^

*ΔE* = [(ΔL*)^2^+(Δa*)^2^+(Δb*)^2^]^1/2^

### 2.6 Determination of water content, water activity, and pH of whale meat

The water content of whale meat was measured using a Halogen Moisture Analyzer (HG63, Mettler Toledo, Zurich, Switzerland); 3 g of each sample (raw whale meat) was heated at 137 °C. The water content was calculated using the following equation:

Water content (%) = (Ws − Wd)/Ws × 100, where Ws is the weight of raw whale meat and Wd is the weight of dried whale meat.

The water activity of whale meat was determined simply using a water activity meter (MD-AW, AS ONE Corporation, Osaka, Japan) at room temperature (25 °C). The pH of whale meat was measured using a pH meter (F-72, Horiba, Kyoto, Japan) equipped with a needle-type electrode (6252-10D, Horiba).

### 2.7 Sensory evaluation

Sensory evaluation was performed on three whale meat samples (before deep-sea storage and after deep-sea storage at 4000 m and 6000 m for 4 months) by 15 trained panelists (9 males and 6 females; 20–40 years of age; students and staffs of the Faculty of Agriculture, Niigata University, Japan). Each sample (200 g) was heated in a hot water bath at 80 °C for 30 min, cut into pieces of 20 × 15 × 10 mm, and then served at room temperature (20 °C). The three different samples were coded and served in a randomized order. The samples were evaluated with a scoring system. Texture (tenderness, looseness of fiber, ease of chewing off, juiciness, and low residue) was evaluated as analytical type test (weak to strong or little to much), and appearance and overall taste were assessed as palatability test (poor to good) using a 7-point balanced semantic scale. Panelists were provided with a cup of water to refresh their palate after testing each sample.

### 2.8 Statistical analysis

Statistical analyses were performed using Excel 2010 (Microsoft, Redmond, WA, USA) with the add-in software Statcel 3 (OMS, Saitama, Japan). The statistical significance of differences between two sets of data was analyzed using Student's *t*-test. Differences between more than two sets of data were assessed using one-way analysis of variance followed by Tukey's multiple comparison test. The significance level was set at *p* < 0.05. All experiments were conducted in at least triplicate.

## 3 Results and discussion

### 3.1 Microbiological safety of whale meat during deep-sea storage and refrigerated storage

First, the contamination and growth of microorganisms in whale meat after storage in the deep sea were examined. The storage temperature of whale meat during deep-sea storage remained constant at 1.6 ± 0.1 °C regardless of water depth and location. First, the change in the number of total aerobic bacteria in whale meat during storage at a depth of 2200 m (22 MPa) in the deep sea was examined. The number of total aerobic bacteria of whale meat before and after 2 weeks, 3 months, and 4 months of storage were 1.2 ± 0.3, 1.8 ± 0.7, 4.1 ± 1.3, and 4.4 ± 0.1 log CFU/g, respectively. After 4 months of storage, the number of aerobic bacteria in whale meat stored at atmospheric pressure was approximately 10^7^ CFU/g, whereas the aerobic bacterial counts in whale meat stored in the deep sea was approximately 10^4^ CFU/g. The difference in the bacterial counts between deep-sea stored whale meat and control increased as storage time increased. Thus, in whale meat stored in the deep sea, the pressure tended to inhibit the growth of aerobic bacteria compared with the control stored at atmospheric pressure; however, they gradually grew with the storage period. Since bacterial cell division started to be inhibited under a pressure of 20 MPa [34], similar inhibition was assumed to occur in this study, and the aerobic bacterial counts were also reduced when compared with storage at atmospheric pressure. From the results of this study, the contaminating bacteria of whale meat can be inhibited by long-term storage under pressure, delaying the spoilage, although bacterial growth was observed even under pressure at a depth of 2200 m in the deep sea.

**Fig. 1:**
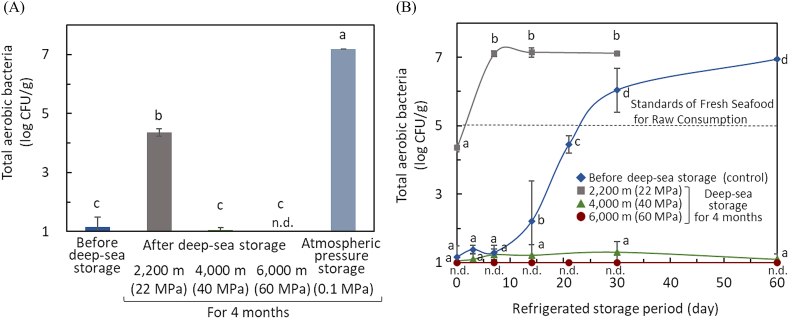
Changes in the aerobic bacterial counts of whale meat before and after deep-sea storage and during refrigerated storage Aerobic bacterial counts of whale meats stored under atmospheric pressure and pressure conditions at depths of 2200, 4000 and 6000 m in deep sea for 4 months were determined by the viable plate count method. A, Effect of pressure on total aerobic bacteria in whale meat after 4 months of storage at depths of 2200–6000 m in deep sea; B, Changes in aerobic bacterial counts of whale meat stored at depths of 2200–6000 m in deep sea during refrigerated storage at 4 °C after deep sea storage. Different letters (A) or different letters in the same storage at depth of deep sea (B) show a significant difference (*p* < 0.05; *n* = 3). n.d., not detected.

Since the above results suggest that the contaminating bacteria in whale meat was inhibited during the storage under pressure in the deep sea, the effect of long-term pressure on these bacteria was then examined during the storage of whale meat under pressure conditions at different depths. Fig. 1A shows the effect of pressure on aerobic bacteria in whale meat after 4 months of storage in the deep sea. As the pressure increased with depth, aerobic bacteria were inhibited. In particular, almost no bacteria were detected in whale meat stored at a depth of 4000 m (40 MPa), and the number of bacteria was below the detection limit at a depth of 6000 m (60 MPa). From the results of the coliform and *V. parahaemolyticus* tests, these bacteria were not detected in all samples of whale meat before and after deep-sea storage. Although preliminary tests for anaerobic bacteria on whale meat stored at depths of 4000 and 6000 m were also conducted since whale meat was vacuum-preserved during long-term storage, anaerobic bacterial counts were below detection limits.

In raw meat such as beef and pork, and salmon, aerobic bacterial counts decreased approximately 10^0^^.5–2^ CFU/g and 10^0^^.5–1^ CFU/g after 60 and 50 days at 10 °C and 60 MPa, respectively [26,35]. Although the storage periods and contaminating bacteria were different, the results of this study were similar to that of previous studies because aerobic bacterial counts were almost below the detection limit after deep-sea storage at depths of 4000 and 6000 m (40 and 60 MPa) in this study. The initial bacterial counts in whale meat were low at approximately 10^1^ CFU/g, and the level of contamination may have affected the sterilization efficiency during pressure storage.

To examine changes in aerobic bacterial counts, whale meats stored for 4 months in deep sea were then subsequently refrigerated under atmospheric conditions for 60 days at 4 °C (Fig. 1B). In whale meat stored at a depth of 2200 m, the bacteria grew during refrigerated storage and reached a maximum population for 7 days. Conversely, in whale meat stored at a depth of 4000 m, although a few bacteria were detected during refrigerated storage, bacteria did not grow even after 60 days. In whale meat stored at a depth of 6000 m, bacteria were not detected at all after 60 days of refrigerated storage. Furthermore, whale meat samples were tested for coliforms and *V. parahaemolyticus* during refrigerated storage. The results were negative for all deep-sea stored whale meat after refrigerated storage.

The synthesis of DNA, RNA, and protein of bacteria such as *Escherichia coli* is inhibited at 50, 58, and 77 MPa, respectively [36]. Although numerous cellular processes are inhibited by pressures <100 MPa, many bacteria survive after exposure to >100 MPa. Some researchers have reported that HHP-treated cells can recover viability during storage [37]. Recovery of bacterial viability may be influenced by the pressure treatment time and pressure level [38]. In this study, the contaminating bacteria in whale meat were injured or killed during long-term pressure storage at a depth of 4000 and 6000 m for 4 months, and no recovery was observed during further refrigerated storage after deep-sea storage. Furthermore, aerobic bacteria did not grow during refrigerated storage of whale meats stored at depths of 4000 and 6000 m, suggesting that even pressure of 40 MPa affected the growth ability of bacteria when exposed for a long period.

In Japan, whale meat is generally distributed frozen, even for raw consumption. Therefore, the results of this study include the effects of freezing on whale meat, which is considered a more practical condition in terms of food preservation and distribution, in addition to long-term storage in the deep sea. On the contrary, *V. parahaemolyticus* is damaged by freezing [39], and freezing during transport and long-term pressurized storage in deep sea may have contributed to the nonexistence of bacteria.

Assuming that whale meat is to be eaten raw, the microbiological standard for frozen fresh seafood for raw consumption in the Food Sanitation Law^3^ (total aerobic bacterial counts, <100,000 CFU/g; negative for coliform and *V. parahaemolyticus* tests) was used as the standard for whale meat samples in this study. The total aerobic bacterial counts in the whale meat were still below the standard value after deep-sea storage for 4 months. In particular, the aerobic bacterial counts in whale meat stored in the deep sea at a depth of >4000 m were still below the standard value even after 60 days of refrigerated storage after deep-sea storage, and no coliforms and *V. parahaemolyticus* were detected. These standards were followed both during deep-sea storage and refrigerated preservation after deep-sea storage and that the safety of whale meat was maintained.

Although the growth of contaminating bacteria on whale meat at a depth of 2200 m was not sufficiently inhibited, long-term storage of whale meat in the deep sea at a depth of >4000 m inhibited the growth of aerobic bacteria, coliforms, and *V. parahaemolyticus* to maintain the safety of whale meat. In addition, since the bacteria in whale meat stored in the deep sea did not grow during the refrigerated storage under atmospheric conditions, the growth of the bacteria can be sufficiently inhibited even in view of the storage and distribution of food. Although the high pressure used in this study during deep-sea storage is relatively low <100 MPa, these results suggest that long-term treatment of food products at even these pressures can significantly inactivate and inhibit the growth of contaminating bacteria.

### 3.2 Physical properties of whale meat during deep-sea storage and refrigerated storage

As the long-term storage of whale meat at a depth of 2200 m (22 MPa) was challenging in maintaining microbiological safety standards, in subsequent tests of physical properties, the physical and chemical properties of whale meat stored for 4 months at a depth of >4000 m were compared with meat that had not undergone storage.

The initial elastic modulus, shear stress, and shear strain rate of whale meat after the deep-sea storage decreased significantly compared with those before deep-sea storage (*p* < 0.05, Table 2). The compressive energy was significantly reduced compared with that before deep-sea storage (*p* < 0.05), although the compressive stress was not different from that before storage. No significant difference was between two different depths, 4000 and 6000 m, in all parameters except for the shear strain rate. The difference in pressure of deep-sea storage in this study slightly affected the toughness of whale meat. When whale meats stored in the deep sea for 4 months were refrigerated at atmospheric pressure for 60 days, no significant difference in any parameters was found compared with those before refrigerated storage.

**Table 2:** Changes in the physical properties of whale meat before and after 4-month deep-sea storage and 2-monthrefrigerated storage after deep-sea storage.

(A)									
Refrigerated storage period (day)	Initial elastic modulus ( × 10^6^ N/m^2^)			Shear stress ( × 10^6^ N/m^2^)			Shear strain rate (%)		
	Before deep-sea storage (0.1 MPa)	4000 m (40 MPa)	6000 m (60 MPa)	Before deep-sea storage (0.1 MPa)	4000 m (40 MPa)	6000 m (60 MPa)	Before deep-sea storage (0.1 MPa)	4000 m (40 MPa)	6000 m (60 MPa)
0	4.75 ± 1.50^aA^	2.10 ± 0.39^aB^	1.79 ± 0.22^bB^	9.56 ± 2.54^aA^	0.97 ± 0.35^bB^	1.40 ± 0.48^aB^	85.69 ± 4.49^aA^	52.72 ± 11.57^bC^	66.53 ± 11.64^abB^
3	2.06 ± 0.52^cA^	1.97 ± 0.53^aA^	2.32 ± 0.67^aA^	5.91 ± 2.00^bcA^	1.33 ± 0.40^bB^	1.50 ± 0.55^aB^	94.18 ± 6.54^aA^	61.93 ± 20.44^abB^	67.85 ± 15.95^bB^
7	2.43 ± 0.77^bcA^	1.71 ± 0.60^aB^	1.85 ± 0.42^abA^	8.45 ± 1.58^aA^	1.14 ± 0.29^abB^	1.45 ± 0.33^aB^	84.56 ± 1.65^aA^	63.94 ± 13.31^abB^	66.24 ± 13.58^abB^
14	1.65 ± 0.29^cA^	1.62 ± 0.47^aA^	1.83 ± 0.65^bA^	5.85 ± 1.12^bcA^	1.42 ± 0.77^aB^	2.28 ± 1.65^aB^	89.15 ± 9.14^aA^	72.53 ± 18.29^aA^	75.45 ± 20.55^aA^
21	3.14 ± 0.33^bA^	2.09 ± 0.69^aB^	2.21 ± 0.53^abB^	7.91 ± 2.80^abA^	1.44 ± 0.38^abB^	1.73 ± 0.54^aB^	93.98 ± 10.76^aA^	80.37 ± 22.39^abB^	84.02 ± 18.27^abB^
30	1.65 ± 0.38^cA^	2.19 ± 0.64^aA^	2.00 ± 0.28^abA^	4.38 ± 1.51^cA^	1.49 ± 0.56^abB^	1.90 ± 0.47^aB^	86.07 ± 12.32^aA^	62.16 ± 11.85^abB^	68.17 ± 12.60^abB^
60	3.16 ± 1.20^bA^	2.18 ± 0.92^aB^	1.79 ± 0.29^bB^	5.64 ± 2.31^bcA^	1.18 ± 0.51^bB^	1.41 ± 0.44^aB^	87.43 ± 11.40^aA^	61.18 ± 11.12^abB^	64.36 ± 2.87^abB^

(B)						
Refrigerated storage period (day)	Compressive stress ( × 10^5^ N/m^2^)			Compressive energy ( × 10^5^ J/m^3^)		
	Before deep-sea storage (0.1 MPa)	4000 m (40 MPa)	6000 m (60 MPa)	Before deep-sea storage (0.1 MPa)	4000 m (40 MPa)	6000 m (60 MPa)
0	19.39 ± 0.31^aA^	19.39 ± 0.54^aA^	19.26 ± 0.45^aA^	4.21 ± 0.17^aA^	3.26 ± 0.40^abB^	3.36 ± 0.23^bB^
3	19.25 ± 0.51^aA^	19.35 ± 0.31^aA^	19.03 ± 0.44^aA^	3.36 ± 0.21^bA^	3.47 ± 0.20^aA^	3.24 ± 0.32^bA^
7	19.38 ± 0.24^aA^	19.36 ± 0.49^aA^	19.29 ± 0.44^aA^	3.55 ± 0.26^bcA^	3.54 ± 0.30^aA^	3.60 ± 0.45^bA^
14	19.22 ± 0.35^aA^	19.35 ± 0.44^aA^	19.34 ± 0.42^aA^	3.54 ± 0.34^cA^	3.40 ± 0.41^aA^	3.23 ± 0.24^aA^
21	19.24 ± 0.34^aA^	19.32 ± 0.27^aA^	19.24 ± 0.88^aA^	3.68 ± 0.15^bcA^	3.29 ± 0.45^bB^	3.23 ± 0.91^bB^
30	19.25 ± 0.34^aA^	19.13 ± 0.42^aA^	19.10 ± 0.42^aA^	3.57 ± 0.21^bcA^	3.29 ± 0.35^abB^	3.22 ± 0.27^bB^
60	19.18 ± 0.41^aB^	19.31 ± 0.54^aB^	19.36 ± 0.44^bA^	3.28 ± 0.22^bcB^	3.18 ± 0.41^abB^	3.65 ± 0.34^aA^

The physical properties of whale meat were measured by shear (A) and compressive (B) tests when whale meat was stored at depths of 4000 and 6000 m in deep sea for 4 months and then at 10 °C for 60 days at atmospheric pressure. Values are presented as means ± SD (*n* = 3). Different lowercase (a–c) and different uppercase (A and B) letters indicate significant differences between the storage period at each tested storage condition and between storage conditions at each storage period, respectively (*p* < 0.05).

As mentioned above, heated whale meat becomes tougher. Since it was considered more important to measure the toughness of whale meat after heating from the viewpoint of its application for cooking and processing, the texture of whale meat was measured after heating. As deep-sea storage reduced the initial elastic modulus, shear stress, and shear strain rate, the whale meat after deep-sea storage felt softer when it was first chewed, and their muscle fibers were easier to chew than those without deep-sea storage. The compressive energy also tended to decrease after deep-sea storage, indicating that deep-sea storage made the muscle fibers of the whale meat easier to loosen.

Meat toughness is affected by the amount and conditions of connective tissue and cross-links, myofibrillar integrity, and intramuscular fat [40]. The tissue structure of whale meat is similar to that of other meats such as beef and pork, and myofibrils and connective tissue change with aging similar to that in other meats, resulting in meat tenderization [41]. In addition, the toughness of whale meat is likely to be affected by aging because of the low lipid and high protein contents in whale meat.^2^ The shear stress and compressive stress of cooked beef were 2.5 × 10^4^–3.6 × 10^6^ N/m^2^ and 2.6 × 10^4^ N/m^2^, respectively, suggesting that whale meat has greater toughness than beef [[42], [43], [44], [45]]. Given that the initial elastic modulus and shear stress also tended to decrease slightly even in whale meat stored for a long period of 2 months at atmospheric pressure in this study, the long-term high pressure made the muscle fibers of whale meat easier to loosen during deep-sea storage in addition to aging due to autolysis, which may have affected its toughness.

Despite several theories on the mechanism of the effect of HHP on the tenderization of meats, one of them is based on the disorganization of the meat structure resulting in tenderization [46]. Others support the theory of the increased release of enzymes and subsequent increased proteolytic activity [47,48], and further alternate mechanisms are postulated to be a combination of structural and enzymatic events [49]. However, since it is known that structural changes in meats do not occur at high pressures below 100 MPa [50], it is unlikely that the former theory is supported under high pressure conditions such as those used in this study. Although few reports on the effects of high pressure <100 MPa on the physical properties of meat for long-term storage, Santos et al. reported that the texture after cooking was improved in beef and pork stored for 30 days at 10 °C and 60 MPa [28]. The large amounts of protein in the cytoplasm between myofibrils in whale meat may also have been effective in improving the toughness of whale meat by long-term aging and high pressure during deep-sea storage.

In general, when meat is stored at low temperatures, shear force values decrease because of aging [51]. In this study, the shear force value of whale meat stored in the deep sea did not decrease after 60 days of refrigerated storage after deep-sea storage, suggesting that sufficient aging occurred during deep-sea storage. Although beef is generally aged for 2–3 weeks at 1 °C-2 °C [52,53], 3 months of long-term refrigerated storage at −1 °C were necessary for optimal beef tenderness in a recent study [54]. Since the optimum conditions for aging differ depending on the type of meat, future studies are needed to determine the pressurized conditions to achieve the optimal tenderness of whale meat.

In this study, the results show that whale meat stored for long periods in the deep sea becomes tender because of prolonged pressure at low temperatures.

### 3.3 Color of whale meat during deep-sea storage and refrigerated storage

**Fig. 2:**
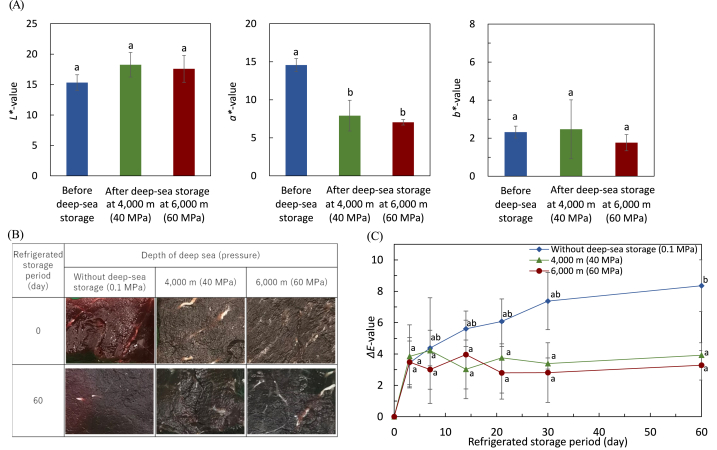
The color and appearance of whale meat before and after deep-sea storage and during refrigerated storage Whale meats stored at depths of 4000 and 6000 m in deep sea for 4 months were refrigerated at 4 °C for 60 days. A, L*-, a*- and b*-values before and after deep-sea storage; B, appearance of whale meat before and after deep-sea storage and refrigerated storage; C, *ΔE* value of whale meat during refrigerated storage. Different letters (A) or different letters in the same storage at depth of deep sea (C) show a significant difference (*p* < 0.05; *n* = 3).

Moreover, the effect of pressure on the color of whale meat after 4 months of storage in the deep sea was examined. Fig. 2A shows the changes in the L*-, a*-, and b*-values of whale meat before and after deep-sea storage. Compared with before deep-sea storage, the a*-value of whale meat after deep-sea storage decreased (*p* < 0.05) although the L*- and b*-values did not change. The *ΔE* values of whale meat after deep-sea storage at depths of 4000 m and 6000 m were 8.23 ± 0.92 and 8.13 ± 0.54, respectively. The C* values of whale meat before and after deep-sea storage at depths of 4000 m and 6000 m were 14.76 ± 0.88, 8.38 ± 2.23 and 7.27 ± 0.39, respectively. C* values decreased after deep-sea storage, indicating a change to a dull appearance. The appearance of whale meat before and after deep-sea storage was also different, appearing black after deep-sea storage (Fig. 2B). Even after 60 days of refrigerated storage, the appearance and *ΔE* of whale meat stored in the deep sea did not change (Fig. 2B and C). No significant difference was noted between the two different depths in color and appearance.

During refrigerated storage, the changes in the color of meats such as beef and pork are manifested as a decrease in lightness (L*-value) and redness (a*-value) [51,55,56]. The decrease in a*-value of whale meat after storage may have contributed to the change in the appearance of whale meat. Even if not under high pressure, long-term refrigerated storage causes oxidation of myoglobin to metmyoglobin and browning. In whale meat without deep-sea storage, *ΔE* increased with refrigerated storage periods at 4 °C, and the color of whale meat was changed because of long-term aging at low temperature. Large amounts of myoglobin are found in more than 5000 mg/100 g of muscle tissue in diving mammals [57]. Whales are one of these diving mammals, and whale meat appears dark red and contains large amounts of myoglobin [58,59]. In this study, the whale meat stored in the deep sea was darker than other raw meats such as beef and pork, suggesting that a large amount of metmyoglobin was produced during storage.

Although the HHP mechanism on color changes has not been clarified, the efficiency of HPP treatment on raw meat color depends primarily on the pressure level, muscle types of meats being pressurized, and initial oxidative state of myoglobin [60]. Normally, the L*-value increases, whereas the a*-value decreases in raw meat after HHP treatment, although the color changes differ depending on the meat type. The meat subjected to HHP treatment of more than 150–300 MPa turned whitish as the pressure increased [61,62]; however, in this study, this was not observed in whale meat stored for a long period at a depth of >4000 m. The appearance of whale meat after long-term pressure storage in this study was different from that of meat products processed by pressure of >100 MPa. In a previous study in which beef and pork were stored at 10 °C and 60 MPa for 60 days, L*- and a*-values remained the same as before storage, whereas b*-values increased [26]. On the contrary, in this study, the L*- and b*-values of whale meat did not change, whereas the a*-value decreased before and after deep-sea storage. Changes in whale meat color were different from the results of beef and pork in a previous study. Thus, changes in the color and appearance of whale meat stored in the deep sea might be affected by high pressure in addition to aging during long-term storage.

### 3.4 Water content, water activity, and pH of whale meat during deep-sea storage and refrigerated storage

The water content and water activity of whale meat did not change before and after deep-sea storage (Table 3). No significant change was found in the water content and water activity of whale meat in refrigerated storage after deep-sea storage (water content, 68.12 ± 1.57; water activity, 0.97 ± 0.01), and after 60 days of storage, water content and water activity were almost the same as before deep-sea storage. It is known that high pressures <100 MPa do not cause mechanical damage to the cellular tissue of vegetables and fruits, or structural changes in meat and fish [50,63]. In a study of beef and pork stored at 10 °C and 60 MPa for 60 days, water content was maintained after storage [26]. The results in the analysis of whale meat in this study were similar to those of other meats. Water activity values of whale meat were within the range of bacterial growth [64], but almost no bacteria were detected in whale meat stored at depths of 4000 and 6000 m. It was suggested that the inhibition of bacterial growth observed in this study was due to pressure during deep-sea storage, not to the effects of water activity.

**Table 3:** Changes in water content, water activity, and pH of whale meat before and after 4-month deep-sea storage and 2-month refrigerated storage after deep-sea storage.

Parameters	Depth of deep sea (pressure)	Refrigerated storage period	
		0 day	60 day
Water content (%)	Before deep-sea storage (0.1 MPa)	64.0 ± 1.2 ^aA^	71.1 ± 1.5 ^aA^
	4000 m (40 MPa)	67.2 ± 1.4 ^aA^	69.1 ± 0.9 ^aAB^
	6000 m (60 MPa)	67.3 ± 1.1 ^aA^	65.9 ± 1.6 ^aB^
Water activity	Before deep-sea storage (0.1 MPa)	0.95 ± 0.01 ^aA^	0.94 ± 0.01 ^aA^
	4000 m (40 MPa)	0.96 ± 0.01 ^aA^	0.97 ± 0.01 ^aB^
	6000 m (60 MPa)	0.97 ± 0.01 ^aA^	0.97 ± 0.01 ^aAB^
pH	Before deep-sea storage (0.1 MPa)	5.56 ± 0.02^aA^	5.59 ± 0.09 ^aA^
	4000 m (40 MPa)	5.61 ± 0.10^aA^	5.68 ± 0.09 ^aA^
	6000 m (60 MPa)	5.54 ± 0.07^aA^	5.47 ± 0.01 ^aA^

The water content, water activity, and pH of whale meat were measured when whale meat was stored at depths of 4000 and 6000 m in deep sea for 4 months and then at 4 °C for 60 days at atmospheric pressure. Values are presented as means ± SD (*n* = 3). Values followed by different small and capital letters in the same column and row are significantly different, respectively (*p* < 0.05).

The pH of the whale meat was approximately pH 5.5–5.6 with and without deep-sea storage. No significant change in the pH of whale meat during deep-sea storage and refrigerated storage after deep-sea storage was observed, and even after 60 days of storage, the pH was almost the same as that before deep-sea storage. The pH of meat generally ranges from 5.0 to 7.0 [65], and the pH of whale meat used in this study was also within this range, although the components of whale meat are different from meat. A previous study of beef and pork stored at 10 °C and 60 MPa for 60 days showed no difference in pH before and after storage [26], and similar results were obtained in this study. Thus, the pH of whale meat was not affected by long-term pressurized and refrigerated storage.

### 3.5 Sensory evaluation of whale meat after deep-sea storage

**Fig. 3:**
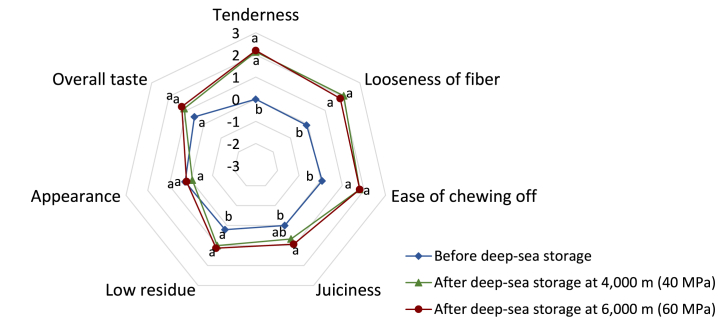
Sensory attributes of whale meat before and after deep-sea storage The sensory evaluation of whale meats stored at depths of 4000 and 6000 m in deep sea for 4 months was conducted using a scoring system. In tenderness, looseness of fiber, ease of chewing off, juiciness and low residue, a 7-point scoring system was conceived as follows: −3, very weak (very little); −2, weak (little); −1, slightly weak (a little less); 0, medium; 1, slightly strong (a little more); 2, strong (much); 3, very strong (very much). In appearance and overall taste, a 7-point scoring system was conceived as follows: −3, very poor; −2, poor; −1, slightly poor; 0, fair; 1, slightly good; 2, good; 3, very good. Each symbol indicates an average score evaluated by 15 panelists. Different letters show a significant difference (p < 0.05; n = 15).

Regarding texture, whale meat stored in the deep sea at 6000 m scored the highest in all categories, except for looseness of fiber (Fig. 3). Significant differences in all texture parameters were observed in whale meat before and after deep-sea storage (*p* < 0.05). The results of sensory evaluation in which whale meat after deep-sea storage at a depth of 6000 m were evaluated to be the most tender, easier to loosen, and easier to chew off were associated with the results of initial elastic modulus, compressive energy, and shear stress assessed by instrumental methods (tenderness and initial elastic modulus, R = 0.997; looseness of fiber and compressive energy, R = 0.999; ease of chewing off and shear stress, R = 0.999). As consumers generally tend to prefer tender meat [66,67], it is considered that the tenderization of whale meat after deep-sea storage in this study was a desirable change. On the other hand, although whale meat before deep-sea storage had the highest appearance scoring, no significant difference was observed in appearance between before and after deep-sea storage samples. Even if the color and appearance of raw whale meat differ before and after deep-sea storage, these differences are expected to be less noticeable after cooking. As this study did not show a significant difference in the overall evaluation of whale meat before and after deep-sea storage, in the future, it will be necessary to conduct preference surveys and examine other factors that constitute taste, such as aroma and flavor in order to improve the overall taste of whale meat. To our knowledge, no previous studies have examined the sensory properties of whale meat. The results of this study indicate that long-term stored whale meat under pressure has become tender enough to be perceived by the human senses as well as physical measurement of texture.

## 4 Conclusion

Although the growth of contaminating microorganisms was observed in whale meat during deep-sea storage at 2200 m (22 MPa), long-term storage of whale meat under pressure and low-temperature conditions in the deep sea at depths of >4000 m (40 MPa) for 4 months was clarified to improve microbiological safety and promote tenderization of whale meat. These effects were also maintained during additional refrigerated storage after deep-sea storage. In the future, other factors will need to be further examined to measure water retention, sensory evaluation of flavor and preference survey of whale meat for general consumers for improvement the quality of whale meat. The results of this study indicate that deep-sea storage technology for the long-term aging of whale meat may be feasible. Accordingly, more practical studies using whale meat and other food products are needed after considering economic aspects such as the cost of deep-sea storage.

## Ethical approval

Review and/or approval by an ethics committee was not necessary for this study because it does not address the ethical considerations of animal, cellular, and human testing. The appropriate protocols for protecting the rights and privacy of all participants were utilized during the execution of the research, e.g. no coercion to participate, full disclosure of study requirements and risks, written consent of participants, no release of participant data without their knowledge, ability to withdraw from the study at any time. Moreover, the products tested were safe for consumption.

## CRediT authorship contribution statement

**Satomi Tsutsuura:** Writing – review & editing, Writing – original draft, Validation, Methodology, Investigation, Funding acquisition, Formal analysis, Conceptualization. **Maki Matsumoto:** Investigation, Formal analysis. **Kana Sakai:** Investigation. **Ryosuke Motegi:** Investigation. **Tadayuki Nishiumi:** Supervision, Project administration, Funding acquisition, Conceptualization.

## Declaration of competing interest

The authors declare that they have no known competing financial interests or personal relationships that could have appeared to influence the work reported in this paper.

## Footnotes

2 https://fooddb.mext.go.jp/index.pl (Dec. 5, 2023) (in Japanese).

## Footnotes

1 http://www.whaling.jp/english/index.html (Dec. 5, 2023).

3 https://www.mhlw.go.jp/stf/seisakunitsuite/bunya/kenkou_iryou/shokuhin/jigyousya/shokuhin_kikaku/index.html (Dec. 5, 2023) (in Japanese).
